# A simplified method for identifying early CRISPR-induced indels in zebrafish embryos using High Resolution Melting analysis

**DOI:** 10.1186/s12864-016-2881-1

**Published:** 2016-08-04

**Authors:** Éric Samarut, Alexandra Lissouba, Pierre Drapeau

**Affiliations:** 1 Department of Neurosciences, Research Center of the University of Montreal Hospital Center (CRCHUM), Université de Montréal, Montréal, QC Canada; 2 Department of Pathology and Cell Biology, Université de Montréal, Montréal, QC Canada; 3 CRCHUM Tour Viger R09.482, 900 rue saint Denis, Montréal, QC H2X 0A9 Canada

**Keywords:** Mutagenesis, CRISPR, Zebrafish, Genotyping, High-Resolution-Melting

## Abstract

**Background:**

The CRISPR/Cas9 system has become a regularly used tool for editing the genome of many model organisms at specific sites. However, two limiting steps arise in the process of validating guide RNA target sites in larvae and adults: the time required to identify indels and the cost associated with identifying potential mutant animals.

**Results:**

Here we have combined and optimized the HotSHOT genomic DNA extraction technique with a two-steps Evagreen PCR, followed by a high-resolution melting (HRM) assay, which facilitates rapid identification of CRISPR-induced indels. With this technique, we were able to genotype adult zebrafish using genomic DNA extracted from fin-clips in less than 2 h. We were also able to obtain a reliable and early read-out of the effectiveness of guide RNAs only 4 h after the embryos were injected with the constructs for the CRISPR/Cas9 mutagenic system. Furthermore, through mutagenesis kinetic assay, we identified that the 2-cell stage is the earliest time point at which indels can be observed.

**Conclusions:**

By combining an inexpensive and rapid genomic DNA extraction method with an HRM-based assay, our approach allows for high-throughput genotyping of adult zebrafish and embryos, and is more sensitive than standard PCR approaches, permitting early identification of CRISPR-induced indels and with applications for other model organisms as well.

## Background

With the development of the Clustered regularly interspaced short palindromic repeats (CRISPR) and CRISPR associated endonuclease 9 (Cas9), fast and reliable genotyping has become an inevitable rate-limiting step for in vivo genomic studies. In zebrafish, commonly used genotyping techniques are based on a locus-specific amplification by PCR followed by amplicon digestion of a specific restriction enzyme or sequencing. This requires adequate genomic DNA for the reaction and substantial time to complete the screen. Recently, new techniques have emerged using fluorescent PCR or high-resolution melting curve (HRM) analysis that simplify and shorten genotyping assays [1–3]. Specifically, HRM assays rely on the high-resolution monitoring of the denaturation process of a double-stranded fluorescently-labeled DNA fragment. This technique can identify micro-indels as small as a single nucleotide and even class-4 single nucleotide polymorphisms (SNPs) [4]. While there are numerous commercial methods for extracting genomic DNA, their price and the fact that they usually require multiple manual steps makes them ill-equipped for high-throughput assessment of indels. Moreover, some genomic extraction methods have been developed such as the HotSHOT method [5] but these have been used in standard PCR amplification.

Here we report the combination of the HotSHOT raw genomic extraction method followed by a HRM analysis for genotyping both adult zebrafish and individual embryos. This optimized protocol allows fast (within two hours) and cost-efficient adult genotyping. Indeed, in our hands, HRM genotyping is more than four times cheaper than regular PCR reaction followed by Sanger sequencing. Furthermore, we also took advantage of this method to identify CRISPR-induced mutations in embryos after microinjection of CRISPR-cas9 constructs. Standard methods generally assess the efficacy of CRISPR/Cas9 mutagenesis by extracting DNA from a 24-h embryo and indels are usually detected by T7E1 endonuclease assay, restriction enzyme screening or fluorescent PCR [6–9]. Our optimized protocol allows the reliable detection of CRISPR/cas9-induced indels in four hour old embryos. Furthermore, we show that our HRM-based identification of indels is much more sensitive than standard sequencing of PCR fragment. Lastly, we took advantage of our protocol to identify the earliest time at which we can detect CRISPR/cas9-induced indels in the embryo. Our results show that CRISPR-induced mutations are detectable as early as after the first cell division of the embryo.

## Results and discussion

### HotSHOT genomic DNA extraction method is suitable for HRM analysis

**Fig. 1:**
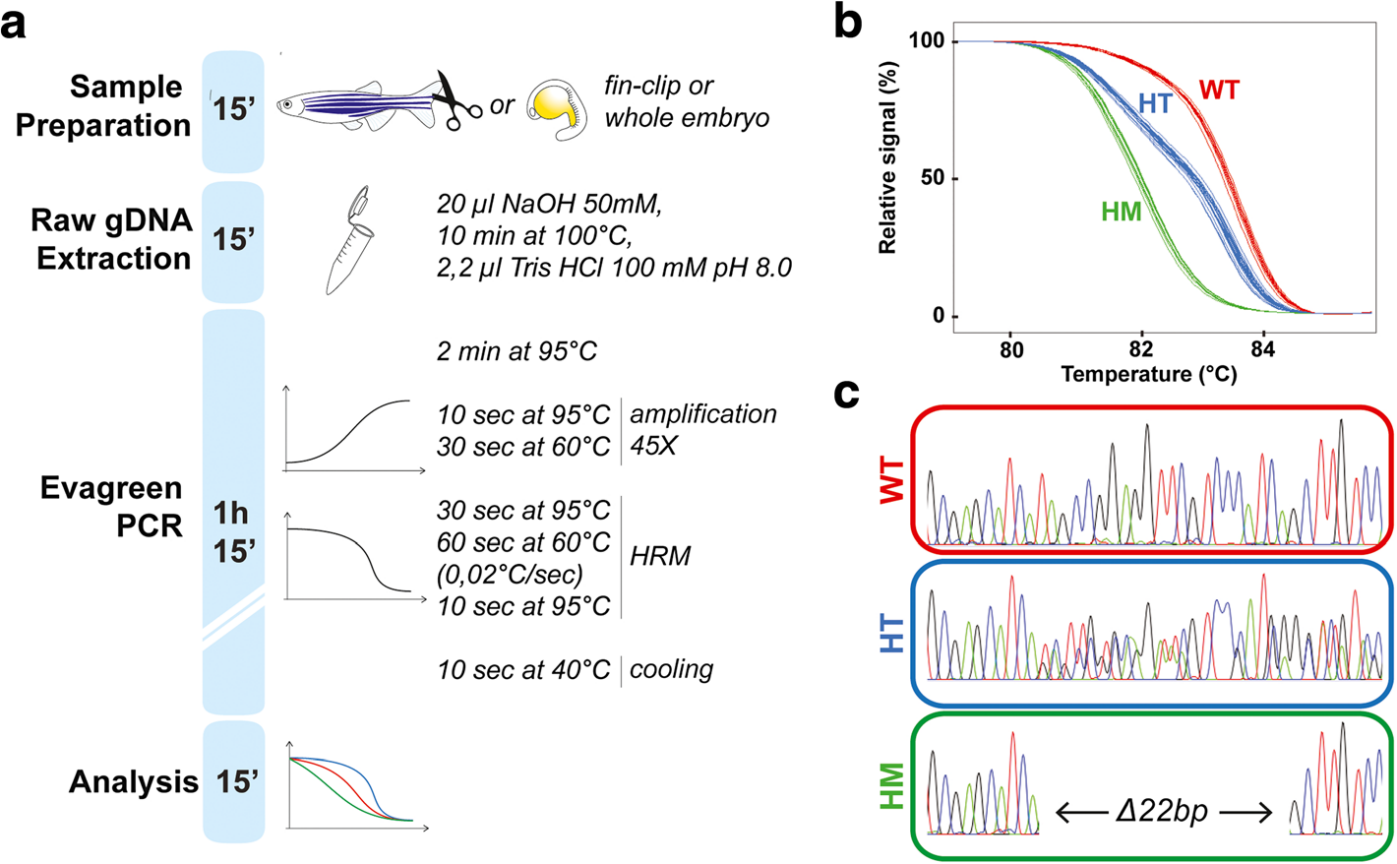
Genomic DNA is suitable for HRM-based genotyping. **a** Protocol timeline for sample preparation. Genomic DNA extraction, two-steps Evagreen PCR and HRM-based analysis. **b** Genotyping of 21 adult zebrafish obtained by an incross of *glra4a* ^*+/−*^ parents. The HRM curve analysis discriminates wild-type (WT), heterozygous (HT) and homozygous (HM) fish. **c** Sequencing results from each profile identified in (B) confirmed the genotyping and the presence of the 22 base-pair deletion in the heterozygous and homozygous population

Our aim was to establish a reliable and cost-effective method for genotyping zebrafish embryos subjected to CRISPR/cas9 mutagenesis that could be used as a precise analysis tool for a broad range of applications. We optimized protocol of raw genomic DNA extraction followed by a 2-steps Evagreen PCR protocol and a HRM analysis. As illustrated in Fig. 1a, our whole genotyping assay could be completed in about two hours. This rapid assessment is particularly important for reducing the time the animal spent in isolated tanks, particularly in laboratories with space limitations. Briefly, genomic DNA is extracted from caudal tail tissue, or from a whole one-day-old embryo by boiling the sample in sodium hydroxide for ten minutes. The high pH of sodium hydroxide is buffered by adding one tenth of the volume of 100 mM Tris HCl pH 8 as described by [10]. Raw genomic DNA is then used as a template in a two-step Evagreen-based PCR reaction in a 96-well plate using a LightCycler 480 device (see material and methods). A final melting step records the fluorescence over an increasing temperature gradient with a high resolution of 0.02 °C per second. We tested this procedure by fin-clipping a heterogeneous population of fish obtained from an incross of two parents carrying a known mutation in *glra4a* gene encoding a glycine receptor subunit, consisting of a deletion of 22 nucleotides (Fig. 1c). Following our procedure, the final melting curve analysis identified three profiles that were confirmed by sequencing to be the expected wild type (*glra4a*^*+/+*^), heterozygous (*glra4a*^*+/−*^) and homozygous (*glra4a*^*−/−*^) genotypes (Fig. 1b, c).

**Fig. 2:**
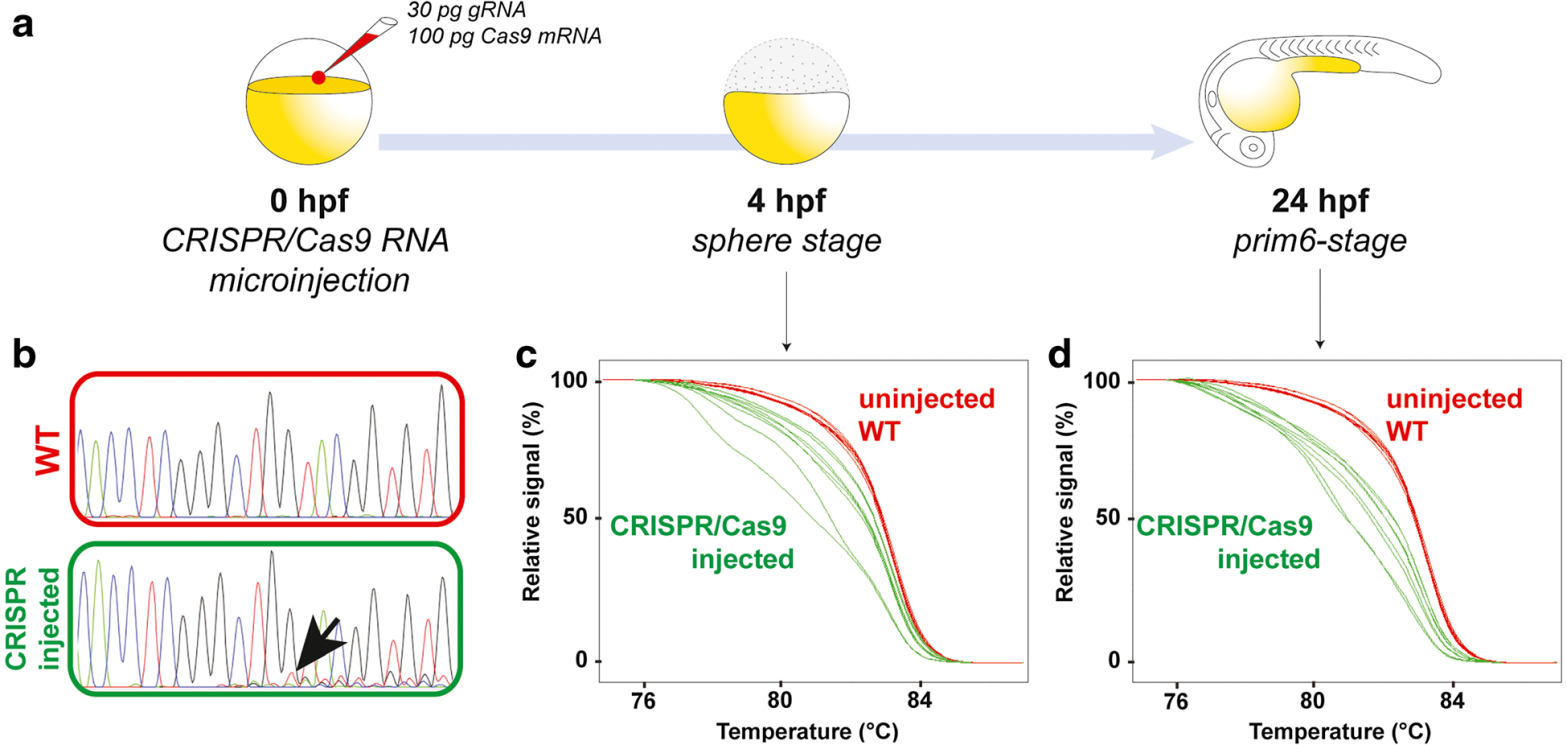
CRISPR-induced indels can be detected in the late zebrafish blastula. **a** RNAs encoding the Cas9 endonuclease and a guide RNA (gRNA) targeting *gldc* coding sequence are co-microinjected into one-cell stage embryos. The genomic DNA was extracted either at the sphere stage (4 h post-fertilization, hpf) or at the prim-6 stage (24 hpf) using the raw extraction method. **b** Sequencing electropherograms of 8 wild type and 8 CRISPR/Cas9-injected embryos at 24 hpf. The black arrow shows the presence of faint multiple peaks in the CRISPR/cas9-injected embryos, indicating the presence of indels. **c** HRM curve analysis of uninjected wild type (WT, red curves) and CRISPR/Cas9-injected (green curves) embryos at 4 hpf. The green curves are shifted and irregular compared to the red WT curves, indicating the presence of indels at the late blastula stage. **d** Melting curve analysis of uninjected WT (red curves) and CRISPR/Cas9-injected (green curves) embryos at 24 hpf

Furthermore, we wanted to see if we could use this method to detect CRISPR-induced mutations directly in the injected embryo. In vitro transcribed RNAs encoding the Cas9 endonuclease and a gene-specific guide RNA (gRNA) are co-microinjected in the one-cell stage zebrafish egg (Fig. 2a). Confirmation of the efficacy of the designed gRNA is crucial for the successful generation of mutant lines. Different techniques are commonly used to detect CRISPR/cas9-induced indels such as PCR sequencing, or restriction enzyme screening but these methods generally assess the efficacy of the mutagenesis one day after microinjection and the results are not always easily interpretable. In fact, detection of indels by PCR sequencing of an injected embryo usually leads to multiple peaks in chromatograms at the indel site and onwards, thus such an analysis might be tricky as it could be easily assimilated as background noise (black arrow, Fig. 2b). To circumvent this problem, new techniques have been developed such as fluorescent PCR or HRM analysis [2, 8, 11].

Thus, we decided to test our HRM-based optimized protocol for the detection of CRISPR/cas9-induced indels within the coding sequence of the glycine decarboxylase *gldc* gene and to confirm that our method allowed the reliable detection of mutations in injected embryos subjected to CRISPR/Cas9-editing (Fig. 2c, d). Indeed, raw genomic DNA extraction followed by HRM analysis (as described in Fig. 1a) from 24 h post-fertilization (hpf) CRISPR-injected embryos led to shifted and irregular melting curves compared to wild type larvae (Fig. 2d). The irregular profiles of these curves are explained by mosaic heteroduplex PCR fragments formed because of the random mutations induced by CRISPR/Cas9 mutagenesis [2]. Moreover, we decided to take advantage of our method to try to detect these indels earlier during development since this would allow a more rapid checkpoint of the mutagenesis efficacy. As shown in Fig. 2c, we successfully identified CRISPR/Cas9-induced mutations in *gldc* by HRM from genomic DNA of a 4 hpf zebrafish blastula. In contrast, at this stage, we were unable to amplify the locus of interest by standard PCR and therefore could not detect the indels by sequencing demonstrating that the HRM-based analysis from a raw genomic extract of a late blastula is more sensitive than standard PCR and allows an early identification of the indels. As a result, this method is very useful to rapidly and accurately assess the efficiency of a CRISPR/cas9 mutagenesis assay in zebrafish.

### CRISPR/CAS9 induces indels in the 2-cell stage embryo

**Fig. 3:**
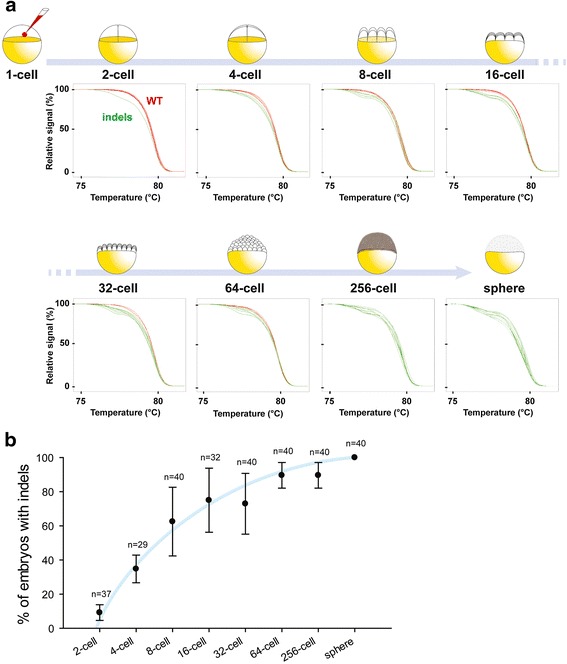
Mutagenesis kinetics of CRISPR/Cas9-induced injected embryos from 2-cell stage until the sphere stage. **a** Melting curve analysis indicated that indels can be detected as early as 2 cell-stage and the proportion of embryos with indels within *calpn1a* coding sequence increases with time (a sample of 8 HRM curves are shown by timepoint). **b** Quantification of the percentage of embryos with indels within *calpn1a* coding sequence at different stages of development (n indicates the number of individual embryos sequenced per timepoint; at least two independent batches of embryos were used per timepoint). The blue line is an approximation of the trendline

Lastly, we decided to go further and try to detect the earliest indels induced by CRISPR/Cas9 system in a third gene: *calpn1a*. To do so, we extracted genomic DNA from early embryos from the very first cell division and onwards until the sphere stage (Fig. 3a). As a result, using our fast HRM assay, we performed mutagenesis kinetics from the very beginning of embryogenesis until the late blastula stage. Interestingly, we were able to detect indels within *calpn1a* coding sequence in embryos as early as the 2-cell stage (just after the first cell division; *n* = 4/37). To our knowledge, this is the earliest time point at which CRISPR/Cas9 mutagenesis has been identified in zebrafish embryos. The percentage of embryos with indels increases significantly during embryogenesis after each cell division and reached 100 % efficiency at the sphere stage (Fig. 3b). This is illustrated by the increasing number of green non-smooth curves during embryogenesis (Fig. 3a, b). This result demonstrates that the CRISPR/Cas9 system is functional very soon after microinjection but that the DNA repair mechanisms are likely actively reversing the majority of induced mutations. However, error-prone none-homologous end joining allowed mutations to occur at the locus of interest (e.g. *calpn1a*) in about 11 % of embryos after the second cell division and this percentage increases to 70 % in 8-cell embryos (Fig. 3b). Interestingly, our quantification suggests that the majority of CRISPR/Cas9-induced mutations arose between the 8-cell and the 32-cell stages during which we observe the maximum variability in the percentage of embryos bearing mutations. This percentage reached a plateau from the 64-cell stage onwards with only a few non-mutated embryos at this stage (*n* = 37/40) to finally reach 100 % of mutant embryos at the sphere stage (*n* = 40/40).

## Conclusions

We propose that the HRM method will expedite genotyping for use with the CRISPR/Cas9 technique. We demonstrated that this method is able to detect CRISPR/cas9-induced mutations from as early as the 2-cell stage embryo and could therefore be useful to develop new CRISPR/Cas9 procedures to reduce the time required to identify mutant embryos., aiming at shifting the mutagenesis plateau even earlier in development. Ultimately, such optimizations would help reduce the amount of mosaicism usually observed in CRISPR-induced mutants. Of note is that our approach should also be feasible for genotyping other genetic model organisms.

## Methods

### Fish husbandry

Wild-type Tupfel long fin (TL) zebrafish (*Danio rerio*) were reared at 28.5 °C, kept under a 12-h dark, 12-h light cycle and staged as described previously [12]. They were bred according to standard procedures [13].

### sgRNA and Cas9 preparation and microinjection

Gene-specific guide RNAs (gRNA) were designed using the online tool CRISPRscan (http://www.crisprscan.org/). We used the following gRNA sequences (lower case indicate mismatches with the genome sequence and PAM site is indicated in brackets): *calpn1a*, gGAGTTCTGGAGTGCCTTGG(TGG); *gldc*, GGGACACCTCGGGCTGGTA(CGG); *glra4a*, GATGCGAGCATCATAGCCGG(AGG). Synthesis of gRNAs and of Cas9 mRNA was performed as described by [14]. Tubingen long fin (TL) wild-type embryos were collected for microinjection. A 1 nL drop of a mix of 100 ng/μL of Cas9 mRNA and 30 ng/μL of gRNA was injected into one-cell stage embryos using a Picospritzer III pressure ejector. These embryos were then used for genomic DNA extraction at different stages of development.

### Fin clipping of adult zebrafish and genomic DNA extraction

Adult fish were anesthetized in tricaine methanesulfonate (MS222) at a final concentration of 160 mg/L and a small piece of the caudal fin was cut with a sharp blade. The fish were immediately put back in fresh water. Genomic DNA extraction was performed in 10 μL (for 2-cell, 4-cell, 8-cell, 16-cell and 32-cell stages) or in 20 μL (all later stages and clipped caudal fin) of 50 mM NaOH from either a clipped caudal fin or from a single whole embryo in its chorion. The samples were boiled for 10 min and 1/10 volume of 100 mM Tris–HCl pH 8 was added to buffer the reaction, as described in [10].

### High-resolution melting (HRM)

Primers were designed using the Universal Probe Library Assay Design Center (Roche). glra4a_for: GCATAAATCCCAAACAAAAGCC; glra4a_rev: CCCCATCGGACTTTCTGG; gldc_for:TTCAGTGAGTATTTGTGTTCTCTACAGG;gldc_rev:TGGTCTGATAGTTGAGTAAGCTCTCC;calpn1a_for:CTTTACCAAAATGTCTATCAGGACG;calpn1a_rev:CGGAGAGCTCATGTTTGTC. All primer sets are available upon request. The PCR reactions were made with 5 μL of the Precision Melt Supermix for HRM analysis (Bio-Rad #172–5112), 0.5 μL of each primer (10 μM) and 2 μL of genomic DNA and water up to 10 μL. The PCR was performed in a LightCycler 480 Instrument II (Roche) using white 96 well plates. Two-step Evagreen PCR reaction protocol was 95 °C for 2 min, then 45 cycles of 95 °C for 10 s and 60 °C for 30 s, followed by 95 °C for 30 s, 60 °C for 60 s, the temperature was increased by 0.02 °C/s until 95 °C for 10 s, then cooling at 40 °C. Curves were analyzed using the Roche LightCycler 480 software version 1.5.1.62.

### PCR and sequencing

Primers were designed using the Universal Probe Library Assay Design Center (Roche). glra4a_for: TGGTTGTTACCAACATCTGG; glra4a_rev: CTGAAATGATTCATGACGC; gldc_for: AATGTATTATTTTTGTGTGAACTGTCCC; gldc_rev: TTCCGTACTTGGCTCTAGTTTGC. All primer sets are available upon request. The PCR reactions were made with 0.5 μL of dNTP (10 μM), 0.5 μL of each primers (10 μM), 2.5 μL of 10x PCR buffer, 0.125 μL of Taq DNA polymerase (GenedireX), 1 μL of genomic DNA and water up to 25 μL. The PCR reaction protocol was 94 °C for 5 min, then 35 cycles of 94 °C for 30 s, 60 °C for 30 s and 72 °C for 45 s and finally 72 °C for 10 min. Samples were sequenced by Genome Quebec/McGill center using Applied Biosystems 3730xl DNA Analyzer.

## Abbreviations

Cas9, CRISPR associated endonuclease 9; CRISPR, Clustered regularly interspaced short palindromic repeats; gRNA, guide RNA; hpf, hours post-fertilization; HRM, high-resolution melting; SNP, single nucleotide polymorphisms

## References

[1] Wittwer CT (2009). High-resolution DNA melting analysis: advancements and limitations. Hum Mutat.

[2] Thomas HR (2014). High-throughput genome editing and phenotyping facilitated by high resolution melting curve analysis. PLoS One.

[3] Vossen RH (2009). High-resolution melting analysis (HRMA): more than just sequence variant screening. Hum Mutat.

[4] Liew M (2004). Genotyping of single-nucleotide polymorphisms by high-resolution melting of small amplicons. Clin Chem.

[5] Truett GE (2000). Preparation of PCR-quality mouse genomic DNA with hot sodium hydroxide and tris (HotSHOT). Biotechniques.

[6] Ota S (2014). Multiple genome modifications by the CRISPR/Cas9 system in zebrafish. Genes Cells.

[7] Auer TO (2014). CRISPR/Cas9-mediated conversion of eGFP- into Gal4-transgenic lines in zebrafish. Nat Protoc.

[8] Ramlee MK (2015). High-throughput genotyping of CRISPR/Cas9-mediated mutants using fluorescent PCR-capillary gel electrophoresis. Sci Rep.

[9] Yu C (2014). A PCR based protocol for detecting indel mutations induced by TALENs and CRISPR/Cas9 in zebrafish. PLoS One.

[10] Meeker ND (2007). Method for isolation of PCR-ready genomic DNA from zebrafish tissues. Biotechniques.

[11] D'Agostino Y (2016). A Rapid and Cheap Methodology for CRISPR/Cas9 Zebrafish Mutant Screening. Mol Biotechnol.

[12] Kimmel CB (1995). Stages of embryonic development of the zebrafish. Dev Dyn.

[13] Westerfield, M. The Zebrafish Book: A Guide for the Laboratory Use of Zebrafish (Danio rerio). Eugene, OR: Univ. of Oregon Press, 1995.

[14] Moreno-Mateos MA (2015). CRISPRscan: designing highly efficient sgRNAs for CRISPR-Cas9 targeting in vivo. Nat Methods.

